# Research on architectural space design based on an empirical investigation of the communication life of older adults: taking unit-type nursing home as examples

**DOI:** 10.3389/fpsyg.2025.1608864

**Published:** 2025-07-08

**Authors:** Hongyi Wang, Ling Zhang

**Affiliations:** Department of Architecture, Soochow University, Suzhou, China

**Keywords:** unit-type nursing home, older adults, communication behavior, communication space, space design of architecture

## Abstract

**Objective:**

This study focuses on unit-type nursing homes for older adults as the research subject, investigating and proposing design methodologies for communication activity spaces that enhance social interaction among older adults.

**Background:**

China’s aging population issue is intensifying, with the number of older adult on the rise. The total count of older adults with disabilities has surpassed 420,000, comprising 16.6% of the entire aging population. The absence of social interaction in unit-type nursing homes can adversely affect the physical health of older adults.

**Methods:**

On-site surveying and mapping of building plans for unit-type nursing homes; Real-time photography of architectural spaces and analysis of spatial design characteristics; Interviews and questionnaire surveys regarding the social needs of older adults; Environmental behavior observation: fixed-point observation of social behaviors and systematic recording of behavioral dynamics among older adults.

**Results:**

A comparative analysis was conducted on the architectural spatial design characteristics of nursing homes with clustered and non clustered architectural spatial layouts. Meanwhile, a comparative analysis was conducted on the social behavior characteristics of older adults living in nursing homes with these two types of architectural spatial composition. Summarize the advantages and disadvantages of architectural design for these two types of space composition in nursing homes. From the perspective of promoting public communication among older adults, propose suitable architectural spatial layout types and communication space optimization design strategies for nursing homes.

**Conclusion:**

The design of living units and small-scale clusters in specialized nursing home should be tailored to the physical health needs of older adults. The architectural space of the nursing home adopts a unit clustered architectural spatial layout type, while the interaction spaces are distributed. This type of building is more conducive to older adults carrying out social activities. It is essential to create a communal communication space within the living unit that embodies an open, family-like atmosphere, serving as the primary venue for both residence and interaction among older adults. Furthermore, urban life functions should be integrated between living units to enhance social communication opportunities for this demographic.

## 1 Introduction

China’s aging population problem is becoming increasingly severe, with the number of the older adults steadily rising. According to data from the Seventh National Population Census, by the end of 2021, the total population of individuals aged 60 and above in China had reached 264.02 million, comprising 18.7% of the country’s total population. Additionally, the total number of the older adults with disabilities surpassed 420,000, accounting for 16.6% of the older adults’ population. In such circumstances, the older adults care problem has attracted the attention of researchers in China (Li and Li, 2012; Zhou et al., 2011). To expand and address these issues, they believe that it is necessary to consider the perspective and content of research on some older adults groups. Previous field investigation and research related to the design of nursing homes can be divided into four different categories according to the content:

1. Studies of new design concept. The research methods include on-site mapping of architectural space, photo shooting, questionnaire surveys and interviews with designers (Bravo-Farré et al., 2012; Liu, 2000; Ma and Zhao, 2004; Feddersen et al., 2009; Huang et al., 2001; Wang et al., 2013).
2. Studies of several typical physical environment space components in nursing home, for example: studies of the public space. The research methods include analyzing the types and design characteristics of architectural space based on the theories and classification methods of architectural typology, analyzing the spatial design features of nursing homes by using architectural space syntax, and conducting post-use evaluation of buildings by applying the methods of environmental behavior science (Zhong et al., 2017; Lu, 2005; Hu, 2004; Xia, 2013).
3. Studies of spatial perception experience in nursing home. The research methods include quantitative evaluation of architectural space photos based on the theory of gestalt psychology, evaluation of the advantages and disadvantages of architectural space design by using the semantic difference method, and evaluation of architectural space experience by combining questionnaires and interviews (Zhou et al., 2013; Xiao, 2017).
4. Studies of carried out from the perspective of the relationship between space and human behavior in nursing home. There are two typical researches among them. The first one discussed the relationship between architectural space design and the behavior pattern of older adults in nursing home (Li and Li, 2012; Zhou et al., 2011). The second one discussed the relationship between architectural spatial elements and behavior types of facilities in institutional nursing homes (Lu et al., 2011). The research methods include architectural space mapping, on-site recording of the behavior types and behavioral trajectories of older adults using environmental behavior science, and assessment of architectural space in combination with questionnaires and interview surveys.

**Figure 1:**
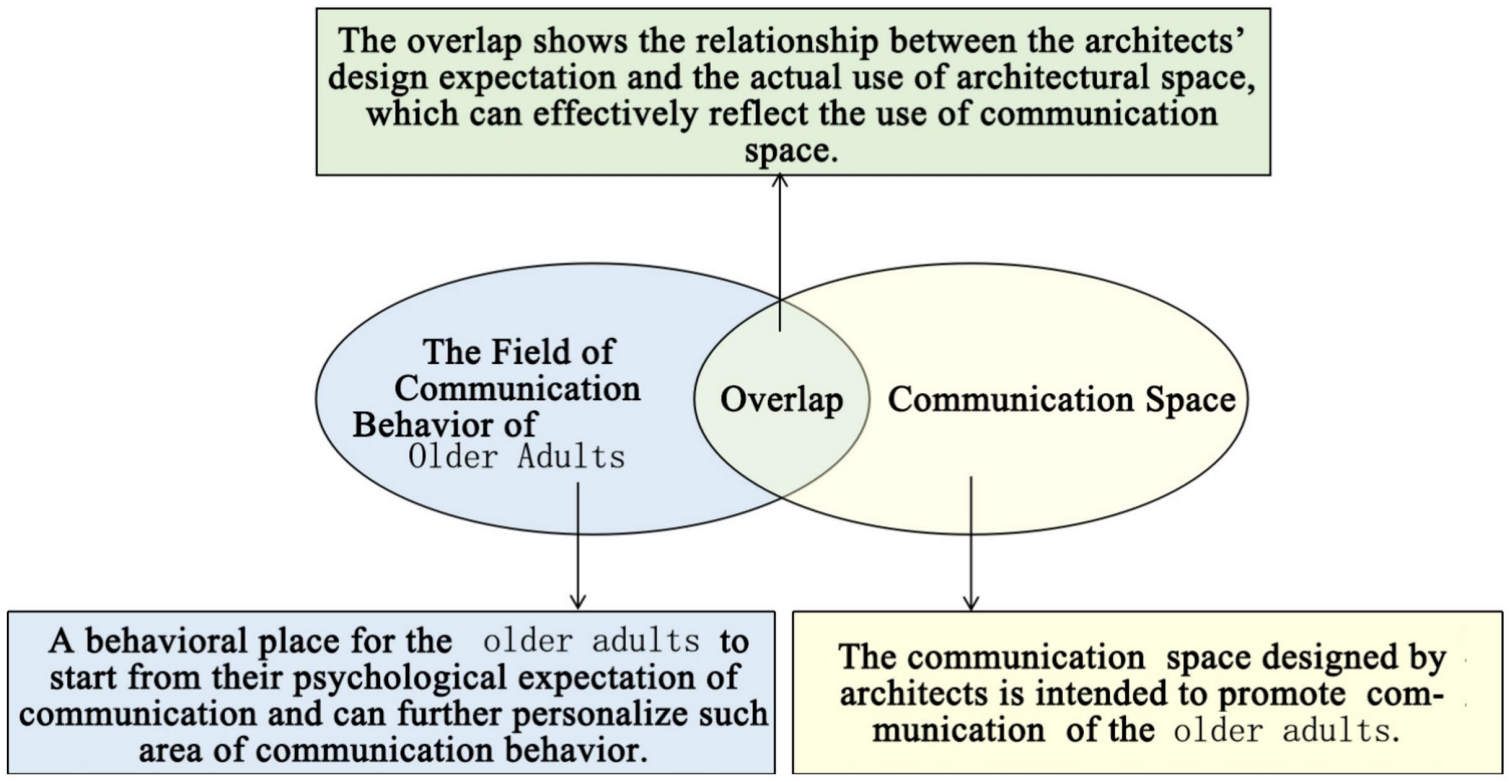
Evaluation of the utilization of communication space in unit-type nursing home.

Based on the previous studies mentioned above, this study innovatively selects two types of nursing homes with clustered and non-clustered architectural spatial layouts as the research objects (Table 1), and takes the promotion of older adults interaction through architectural space design as the research entry point. Meanwhile, the study innovatively applies the theory of mutual penetration between the environment and behavior (Altaian and Rogoff, 1987; Takahashi Takashi and EBS Group, 2006), as shown in Figure 1. Through field investigations, it quantitatively analyzes the overlap degree between the interaction behaviors of older adults and the interaction space of nursing homes, taking the degree of mutual integration between the interaction behaviors of older adults and the interaction space of nursing homes as the research basis. So as to evaluate the advantages and disadvantages of the existing communication space design in nursing home, and a comparative analysis was conducted on the design of nursing homes with clustered and non clustered architectural spatial layout types of nursing units, as well as the influence of decentralized and centralized interaction spatial layouts on the interaction behaviors of older adults; Meanwhile, by using the spatial behavior observation method and questionnaire survey method in environmental behavior science, an empirical investigation and analysis were conducted on the actual communication situation and communication psychological needs of older adults in the nursing home. Furthermore, the theories related to the Psychology of Architecture, environmental behavior and architectural space design are used as theoretical guidance for the design (Takahashi Takashi and EBS Group, 2006; Parke and Friesen, 2015; Zhou et al., 2011), and then an optimization design strategy for the architectural space of the nursing home that effectively promotes the communication of older adults was proposed.

**Table 1:** Basic architectural information and spatial composition of field investigation cases.

Basic information of building		Floor layout of residential buildings for older adults	Types of architectural space composition
DL07	Year of construction: 2010 Building area: 6500 ㎡ Number of beds: 252 Number of older adults: 35 Number of nursing workers: 23	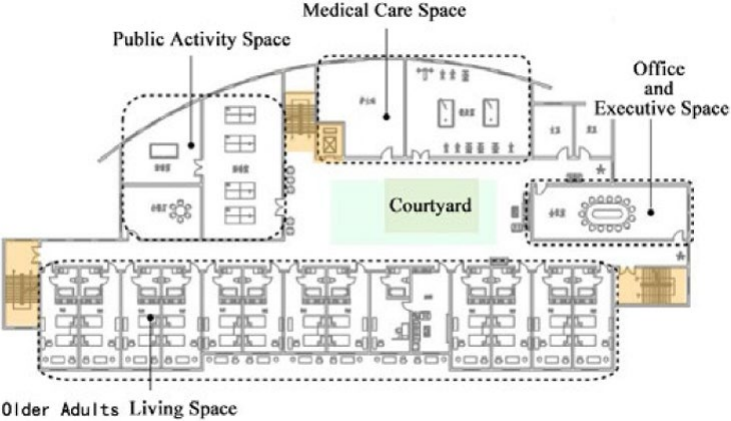	The building adopts a non clustered architectural spatial layout type, and the interaction Spaces are centrally arranged
SY01	Year of construction: 2004 Building area: 2700 ㎡ Number of beds: 100 Number of older adults: 100 Number of nursing workers: 28	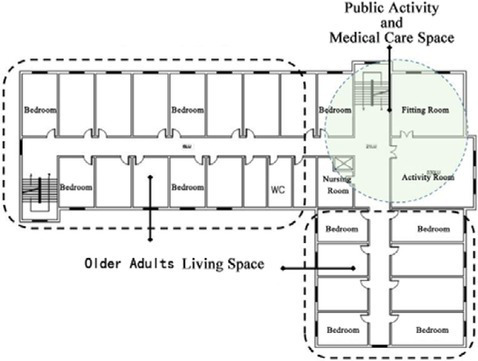	The building adopts a non clustered architectural spatial layout type, and the interaction Spaces are centrally arranged
JP01	Year of construction: 2009 Building area: 1690 ㎡ Number of beds: 100 Number of older adults: 80 Number of nursing workers: 38	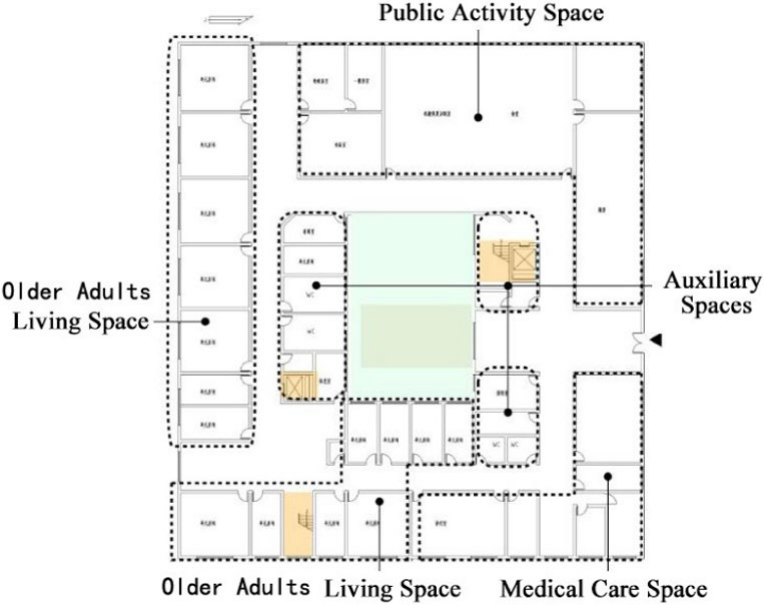	The building adopts a unit clustered architectural spatial layout type, and the interaction Spaces are distributed
JP08	Year of construction: 2012 Building area: 1545 ㎡ Number of beds: 42 Number of older adults: 39 Number of nursing workers: 23	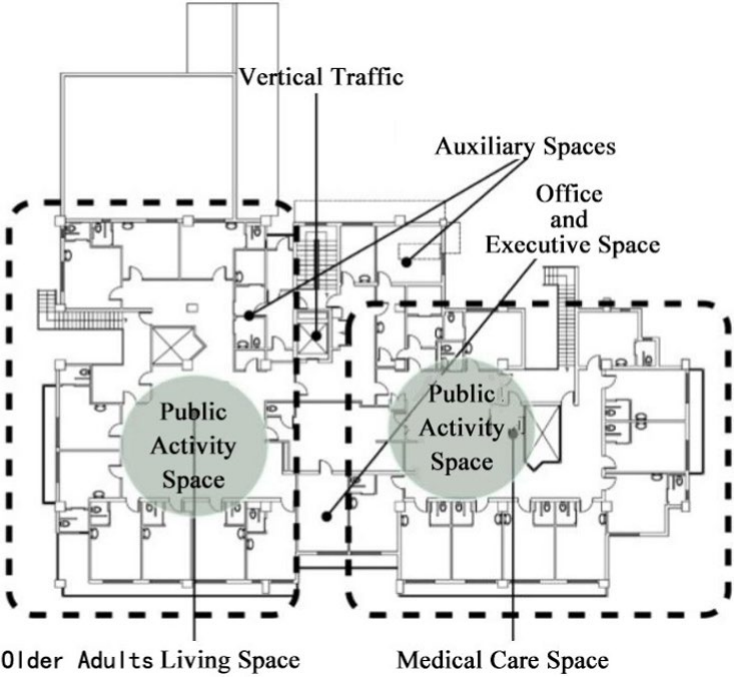	The building adopts a unit clustered architectural spatial layout type, and the interaction Spaces are distributed

At present, older adults who receive professional care in institutions in China have fewer social activities due to their loss of self-care ability. A reduction in social activities may further lead to a deterioration in physical health. If we do not pay attention to this problem and find possible solutions as soon as possible, more of them will be trapped in this vicious circle. Based on this scenario, this study conducts a comparative analysis of the characteristics of older adults’ interaction behaviors, influenced by various building space compositions, through fieldwork conducted in specialized care institutions. Subsequently, it utilizes these findings as a foundation to explore suitable spatial environment design strategies that cater to the psychological interaction needs of older adults, aiming to enhance their mental well-being and, to a certain extent, mitigate their physical decline.

## 2 Method

Based on the relevant data and literature, the spatial type system of the older adults buildings under study and the composition of the welfare policy were systematically summarized. Thus, field studies on the subjects at four levels (environmental observation, behavioral observation, cognitive observation, and questionnaires using architectural planning) were conducted. The interviews were conducted with the managers and older adults residents to understand the basic conditions of the facilities and their feelings about the facilities. After observing the behavioral characteristics of the older adults individuals and their use of each space, relevant research was carried out (Peavey and Vander Wyst, 2017). The research methods shown in Table 2 include questionnaires, plan metric measurements of facilities, illumination measurements and photographs, and recording the walking routes of older adults in key spaces, all of which have great research value.

**Table 2:** Overviews of research methods.

Research methods		Features	Contents
Environmental observation	Investigation into the design	Investigating the design of the older adults building. Mapping the current state of the physical environment of the building. Recording by using drawings, cards, and photographs.	General plan, floor plan, elevation, and section, which were all of certain scale, were used as the basic to record the various elements of the building, and were then analyzed.
	Observation of physical traces	Physical traces are “things that were left unconsciously by the older adults in a older adults building.” Researchers systematically observed the physical environment in order to find traces of previous activities that had not been added for the purpose of measurement.	Researchers inferentially studied the original state of the environment, the use and modification by the older adults for their needs, and the relationship between people and the building environment, by analyzing the physical traces such as residues, wear and tear of public objects, or other aspects without traces.
	Furniture and furnishing observation	The way that the furniture are arranged reveals the potential regularity about space utilization by the older adults and the interaction between space and human behavior.	Living conditions of the older adults and the way to use the space were inferred based on the actual arrangement of furniture in the floor plan.
Behavioral observation	Behavioral plane survey	Observing the relationship between space and behavior. Exploring the suitable state of the older adults building space. Looking for evidence that can help promote activities of older adults individuals.	Marking activities of the older adults (movement, space occupation, behavioral trajectory, cluster behavior, etc.) And relevant issues on the measured floor plan Abstracting the relationship between space usage patterns and behaviors.
	Stationary scene observation	The various behaviors that occur cotemporally in the same behavioral scene at a certain point in time are observed and grasped as stationary scenes.	
	Dynamic scene observation	A series of behaviors that occur over time in the same behavioral scene are observed and grasped as dynamic scenes of “behavioral flow.”	
	Temporal comparison method	The investigation site was set at the same place for observation to capture temporal changes in behavior.	
	Spatial comparison method	The same behavior is observed at several observation sites with different spatial patterns and regional characteristics for the comparison of its spatiality.	
	Behavior tracing investigation	Arguing questions that were raised through preparatory research and analysis of results, and further confirming them through trace investigation.	
Inquiries	Questionnaires	Opinions and requests were asked directly to the older adults living in older adults buildings according to the prepared questions. The question types include objective facts and subjective wishes.	Questionnaires were distributed, filled out and collected
	Interviews	Direct question-and-answer survey were carried out, followed by transcription, including elemental recall method and focus interview method.	Interviews with leaders, facility managers, nursing staff and resident older adults were conducted to learn about the basic conditions of the facilities and how older adults feel about them.

This study is grounded in the theory of mutual infiltration between the environment and behavior (Altaian and Rogoff, 1987; Takahashi Takashi and EBS Group, 2006), as shown in Figure 1. This theoretical framework posits that by observing interaction behaviors within architectural spaces, a higher overlap rate between the interaction behaviors of older adults individuals and the designated interaction spaces in nursing homes indicates a greater utilization rate of these spaces. From an architectural design and spatial layout perspective, such interaction spaces are effectively designed to meet the communication needs of older adults. Conversely, if behavioral observation records reveal that older adults individuals do not engage in interactions within these designated areas, resulting in a lower overlap rate between their behaviors and the nursing home’s interaction spaces, it suggests that these areas are underutilized. In this context, it can be inferred that the architectural design and spatial configuration fail to adequately address the social engagement needs of residents (Altaian and Rogoff, 1987; Takahashi Takashi and EBS Group, 2006). To investigate this phenomenon further, this study conducted field investigations on older adults interaction behaviors across four different nursing homes utilizing relevant environmental behavior research methods (Takahashi Takashi and EBS Group, 2006). Drawing upon the theory of mutual infiltration between environment and behavior, we employed both qualitative observations from field studies and quantitative analyses as metrics for assessing the degree of overlap between older adults interactions and designated interaction spaces. These findings also serve as evaluative criteria for determining the quality of interaction space design within nursing homes.

The specific research process and quantitative analysis are outlined as follows: The researchers conducted observations of the interaction behaviors exhibited by older adults residents in a nursing home over a total period of 3 days. Observations were carried out daily from 8:00 a.m. to 12:00 p.m., and again from 2:00 p.m. to 9:00 p.m. During these observation periods, the researchers systematically recorded the spatial locations where interactions occurred at fixed intervals of every 10 min on an architectural floor plan. This approach yielded one behavioral observation sample per interval. These samples were subsequently superimposed and plotted onto the architectural plan of the nursing home (Table 3), while simultaneous quantitative analyses were performed (Table 4). As illustrated in Table 3, the pink areas on the architectural floor plan denote spaces designated for interactions within the nursing home; conversely, the gray circular regions represent areas where interaction behaviors among residents occurred, derived from overlaying and mapping out observed behavior samples. Additionally, numerical indicators have been included to reflect the frequency of these interaction behaviors among older adults individuals.

**Table 3:** Investigation of the actual state of the communication behavior of older adults.

Research Cases-non clustered nursing units	
DL07 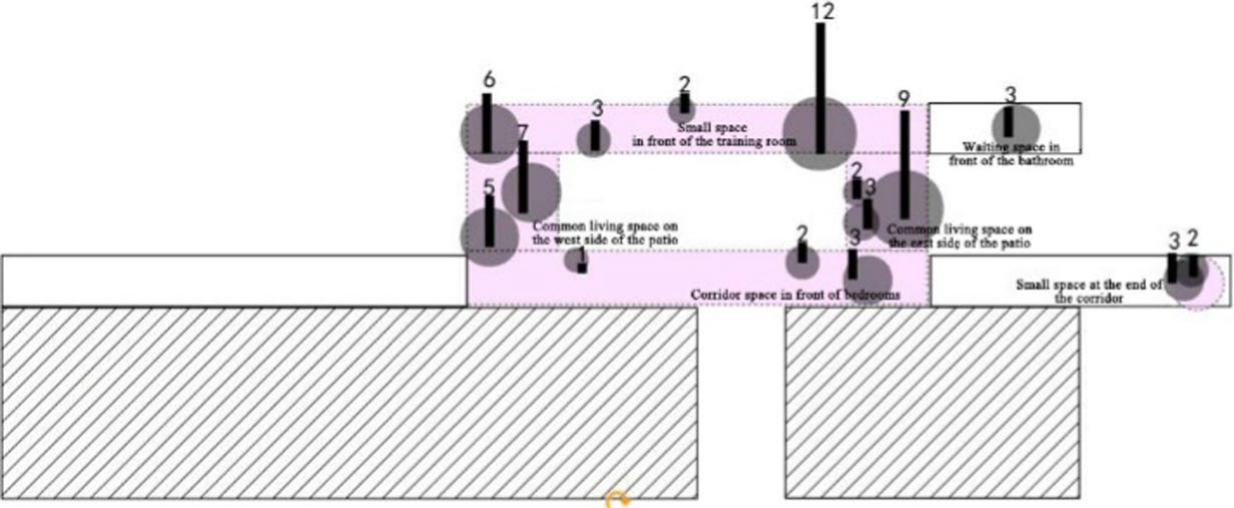	SY01 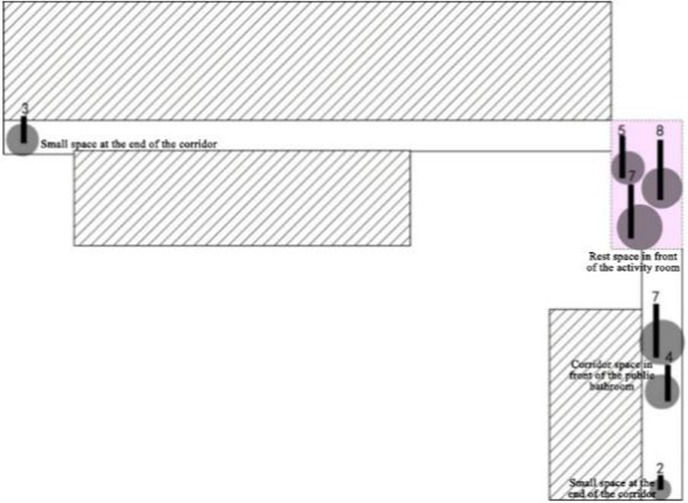

Research Case-group type nursing units	
JP01 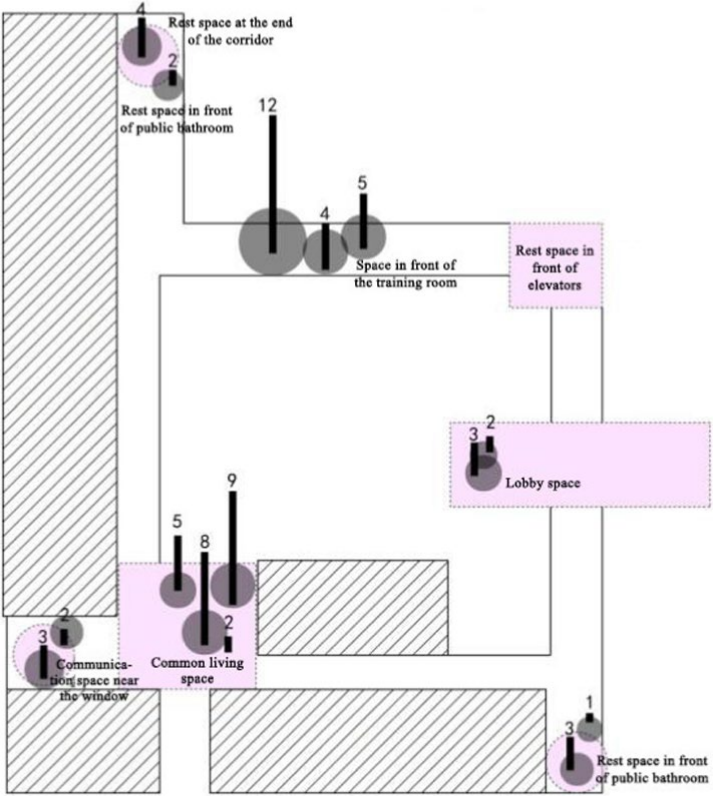	JP08 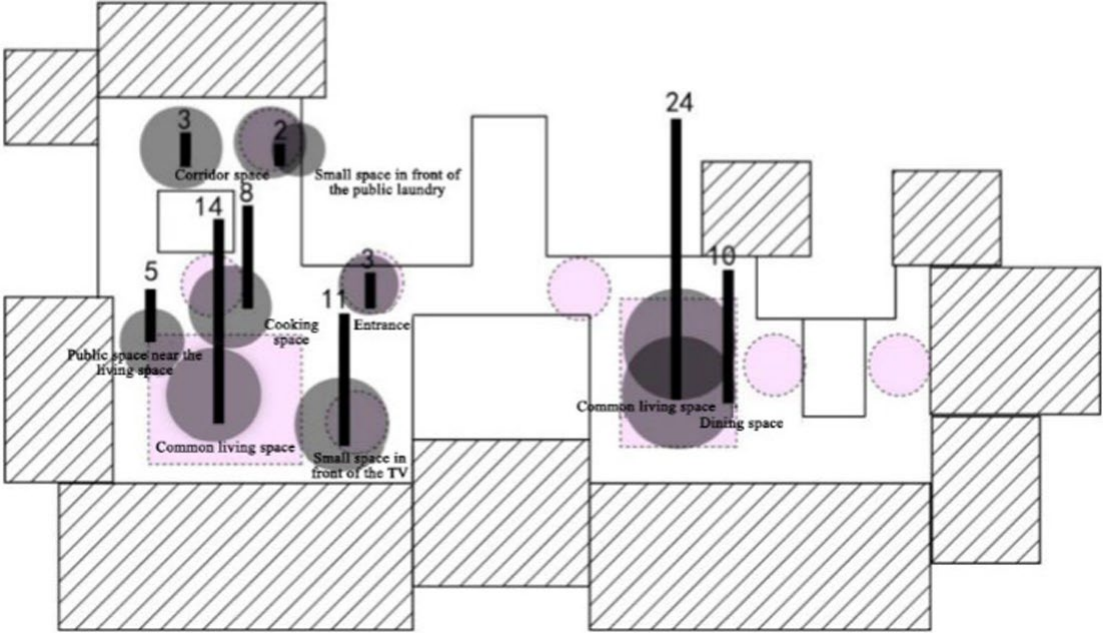
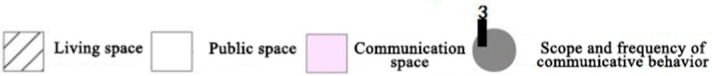	

**Table 4:** Statistics on the overlap and formation frequency of communication behavior fields and communication space of older adults.

Research cases-non clustered nursing units		Research cases-group type nursing units	
DL07		JP01	
The overlap between the field of communicative behavior and communicative space	The formation place and frequency of the field of communicative behavior	The overlap between the field of communicative behavior and communicative space	The formation place and frequency of the field of communicative behavior
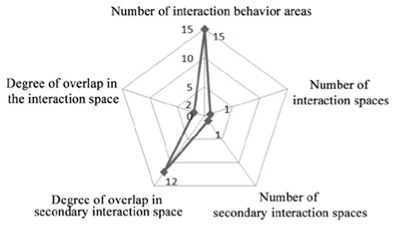	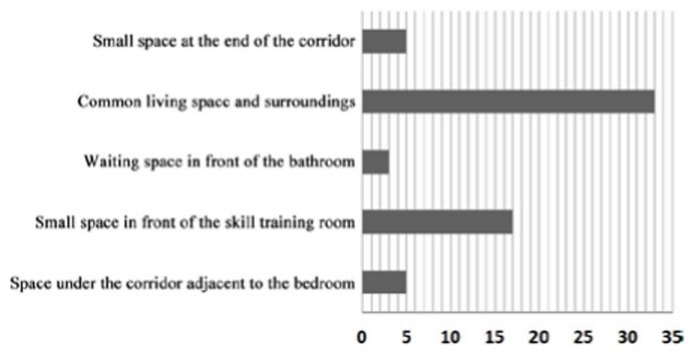	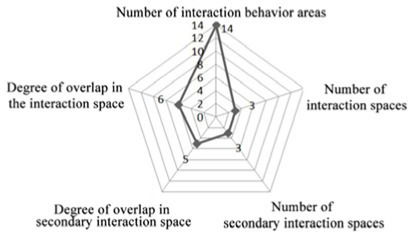	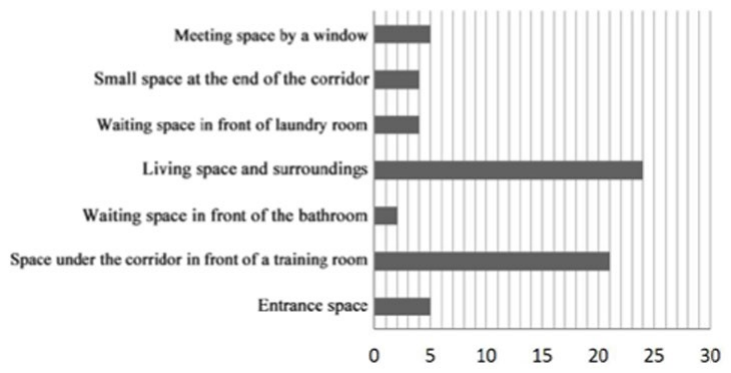
SY01		JP08	
The overlap between the field of communicative behavior and communicative space	The formation place and frequency of the field of communicative behavior	The overlap between the field of communicative behavior and communicative space	The formation place and frequency of the field of communicative behavior
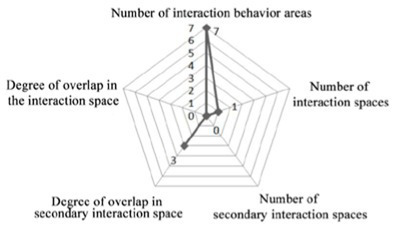	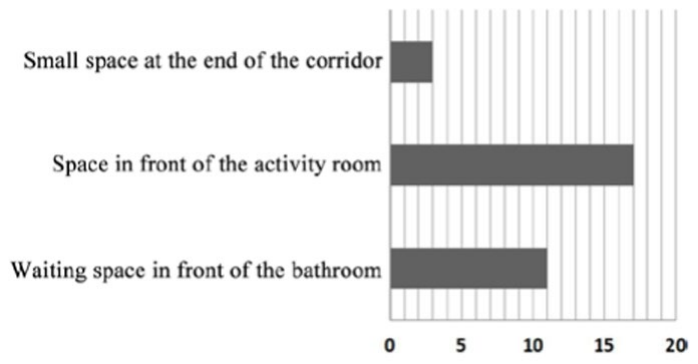	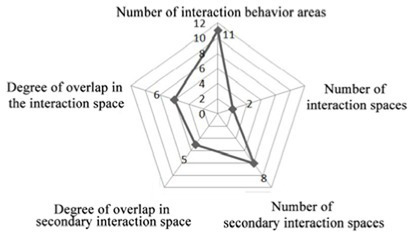	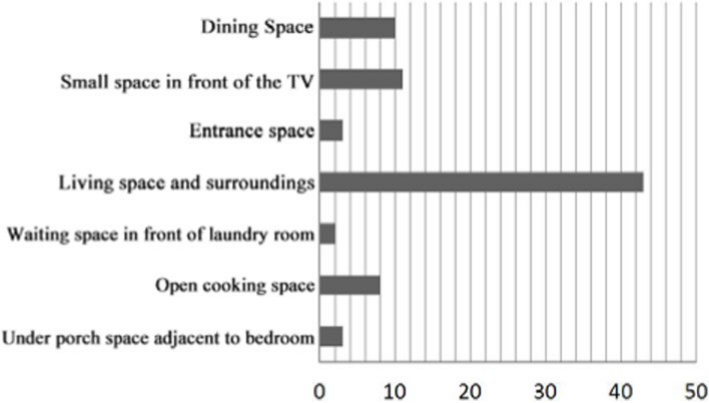

According to the theory of mutual infiltration between the environment and behavior, a higher degree of overlap between the gray circle area (The areas where interaction behaviors among older adults occurred) and the pink area (The existing communication space in the nursing home) in architectural drawings indicates a stronger alignment between older adults interaction behaviors and available interaction spaces. Consequently, this increased overlap correlates with a greater willingness among older adults residents to participate in interactive activities within these designated areas. The design of communication spaces within nursing homes effectively addresses the communicative needs of older adults. In architectural representations, if the gray circle area (The areas where interaction behaviors among older adults occurred) does not fall within the confines of the pink area (The existing communication space in the nursing home), it suggests that older adults individuals have opted not to engage in social activities within existing communal settings. This scenario implies that current designs for social spaces may inadequately meet the needs and preferences of older adults.

## 3 Discussion

### 3.1 Summary of field investigations of unit-type nursing home for older adult

#### 3.1.1 An overview of the nursing homes through on-site investigation and research

The field research was divided into two parts. The first part of the survey collected basic information about the building, including the building plan, the image of the spatial environment in the building, and information about the construction time, the building area, the number of beds, the number of older adults, the number of nursing staff, the content of care, and the utilization of facilities. After that, the characteristics of the architectural space of the research cases were analyzed and summarized. In addition, interviews and questionnaires were used to further understand the interaction needs of older adults. The second part of the survey involved observing and recording the interaction behavior of older adults in special care nursing institutions. The characteristics of the interactions between older adults were analyzed by using the environmental behavior research method. The four nursing homes selected in this study have the same level of medical care functions. This type of nursing home specifically provides living and medical care services for the disabled and semi-disabled older adults. There are differences in the architectural space composition forms of the four older adults care institutions. Among them, the architectural designs of Case DL07 and case SY01 adopt a non-clustered architectural space layout form. The architectural designs of Case JP01 and Case JP08 adopt a clustered-based architectural spatial layout form, as shown in Table 1. The objective of this study is to investigate the daily interaction behaviors of the older adults living in four nursing homes, analyze the influence of the non-grouped architectural spatial layout form or the grouped architectural spatial layout form adopted by the nursing homes on the interaction behaviors of the older adults, and then put forward suggestions for the future optimization design of nursing homes in terms of architectural spatial layout and interaction space design. Table 1 summarizes the basic architectural information and the spatial composition of the buildings in the field research cases.

#### 3.1.2 An overview of the older adults participating in the investigation and research

The older adults who participated in the questionnaire survey and behavioral observation resided on the same floor of the nursing home. All participants were aged 65 years and above, requiring wheelchairs for mobility assistance. None exhibited major health issues, and there was minimal variation in their overall health conditions. In each nursing home, a total of 20 older adults took part in the questionnaire survey and behavioral observation, with ten individuals selected from both the east and west sides of the building on that floor. This sample represented 90% of the residents living on that particular floor. In total, 80 older adults across four nursing homes were involved in this research study. The gender distribution among respondents within each care institution was characterized by a ratio of 4:6 (male to female). For instance, numbers ranging from 01 to 10 indicated those older adults participating in the questionnaire survey and behavioral observation; specifically, these comprised ten individuals residing on the east side of the building as well as another ten living on its west side, all contributing to this research effort.

### 3.2 Analysis of the interaction needs of older adults in field research cases

The relevant theories such as architectural psychology (Takahashi Takashi and EBS Group, 2006), environmental behavior science and architectural space design (Parke and Friesen, 2015; Zhou et al., 2011) are used as the theoretical guidance for the questionnaire on the psychological needs of communication among older adults in the nursing homes. Based on the above theory, questionnaires were made in the four aspects of form, function, location and scale of the communication space design. Then the interviews and questionnaires were used to analyze the intrinsic psychological needs of older adults (Table 5). Through the field study, the following characteristics of the needs of older adults regarding interaction space were identified.

**Table 5:** Investigation of the internal communication psychological needs of older adults in field investigation cases.

Form of communication space preferred by older adults	Functions of communication space
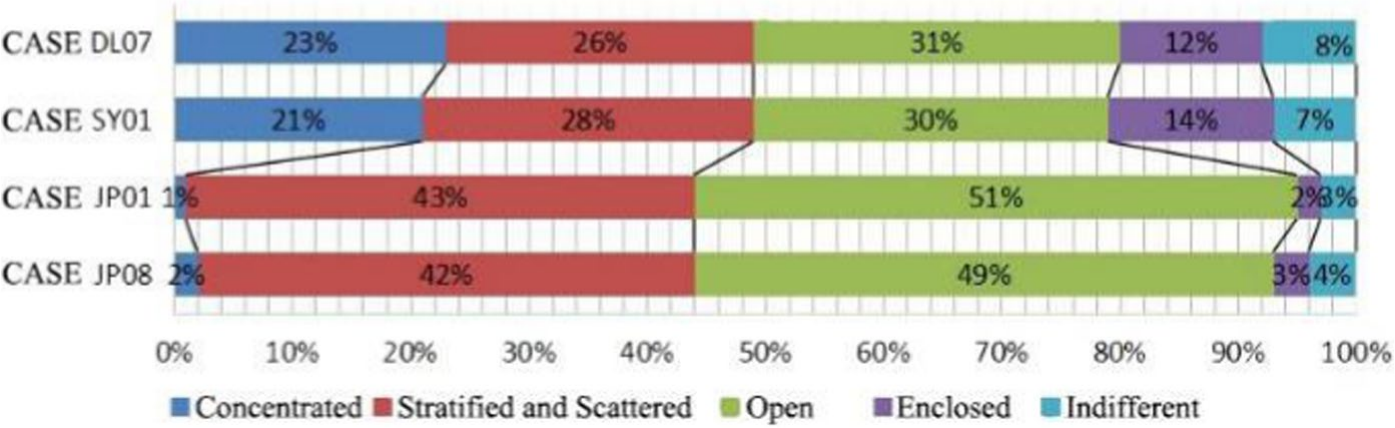	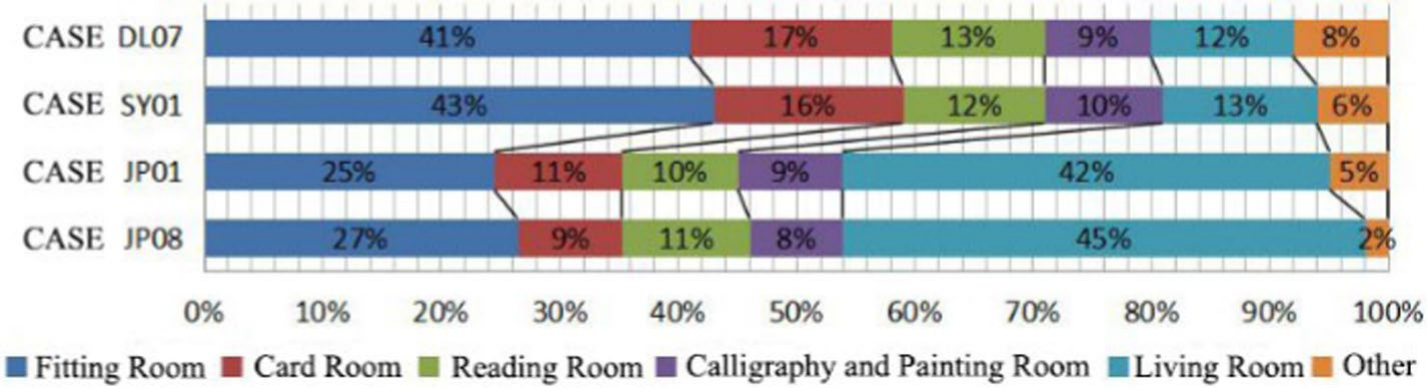

Expected location of the communication space	Adequacy of the existing communication space
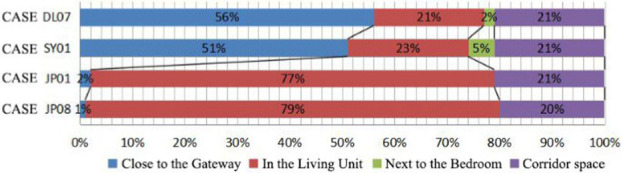	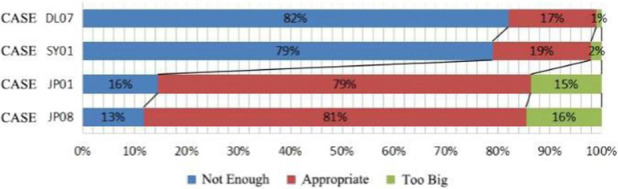

#### 3.2.1 Form of interaction space

Space with stratified, decentralized and open characteristics was desired by older adults.

#### 3.2.2 Function of interaction space

Older adults had a greater demand for recreational interaction space in non clustered nursing units, while older adults had a greater demand for a home-like atmosphere and living function in group type nursing units.

#### 3.2.3 Location of interaction space

All older adults wanted interaction space in the corridor. In addition, the older adults also showed the desire to have interaction space designed in non clustered nursing units.

#### 3.2.4 Size of interaction space

The majority of older adults thought that the space was insufficient and could not meet their daily interaction needs in non clustered nursing units. However, older adults thought that the size was more appropriation group type nursing units.

### 3.3 Investigation of the interaction life of older adults in a field study case

The field investigation includes two aspects: the object of interaction behavior and the domain of interaction behavior.

#### 3.3.1 Investigation and characterization of older adults’ interaction behavior

Through the observation and recording of the communication behavior of older adults in field investigation cases, the communication places often used include bedrooms, shared living spaces, public conversation rooms, functional training rooms, corridor spaces and public rest spaces outside the group unit. In addition, there are differences in the choice of interaction objects between different places in space (Gobbens and van Assen, 2018). The bedroom space includes older adults and their roommates, older adults and their relatives and friends, and older adults and nursing workers. The bedroom space includes older adults and their roommates, older adults and their relatives and friends, older adults and visitors, and older adults and nursing workers. Moreover, the communication modes between different communication objects in unit-type nursing home can be divided into two types: transient communication and continuous communication (Table 6). A scatter plot of the relationships of the interaction objects on the same floor, which was based on field research of behavior observations of cases DL07, SY01, JP01 and JP08, was drawn (Table 7). Through graphic analysis, the following conclusions can be drawn.

1. Scatter plot characteristics of the relationships between communication behavior objects on the same floor based on behavior observations in non clustered nursing units.

**Table 6:** Survey of communication behavior objects of older adults.

Greetings between older adults	Continuous communication between older adults	Greetings between older adults and nursing workers
Continuous communication between older adults and nursing workers	Communication between older adults and relatives and friends	Communication between older adults and non relative visitors

**Table 7:** Scatter plot of communication behavior object relationships of older adults.

Research Cases-non clustered nursing units	Research Cases-group type nursing units
DL07 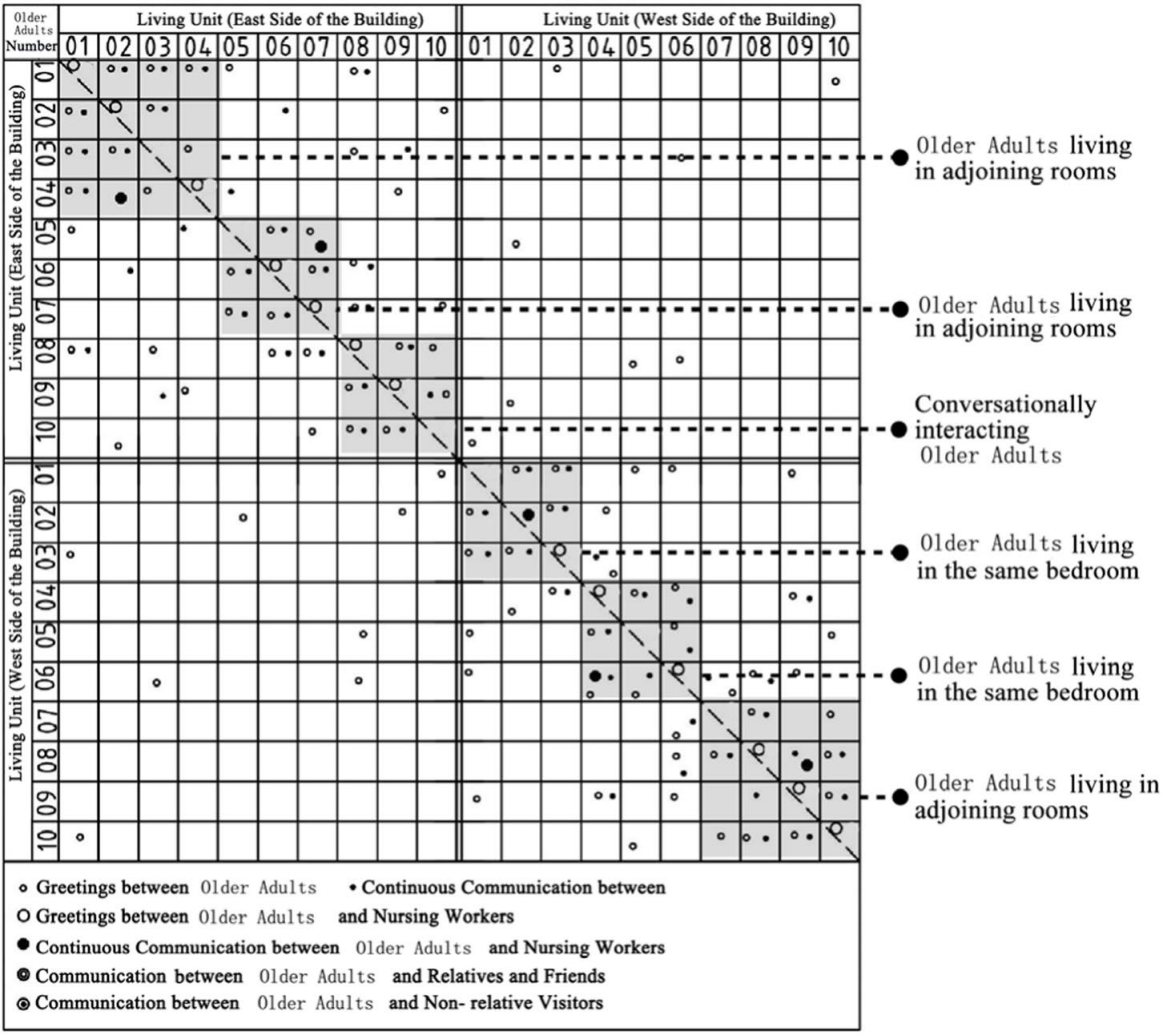	JP01 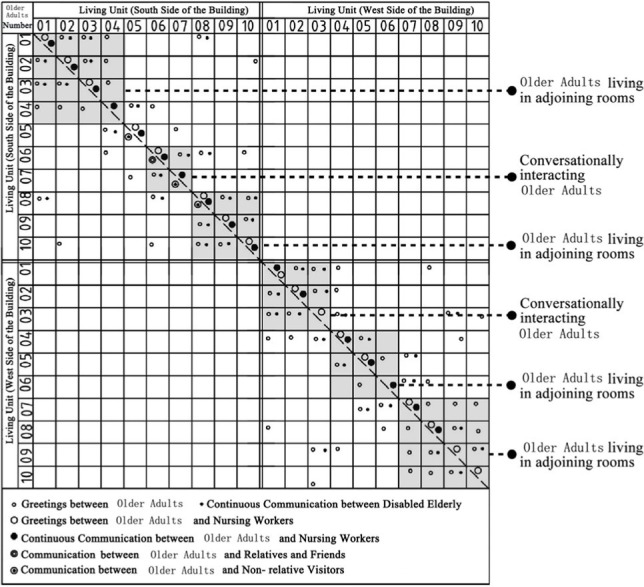
SY01 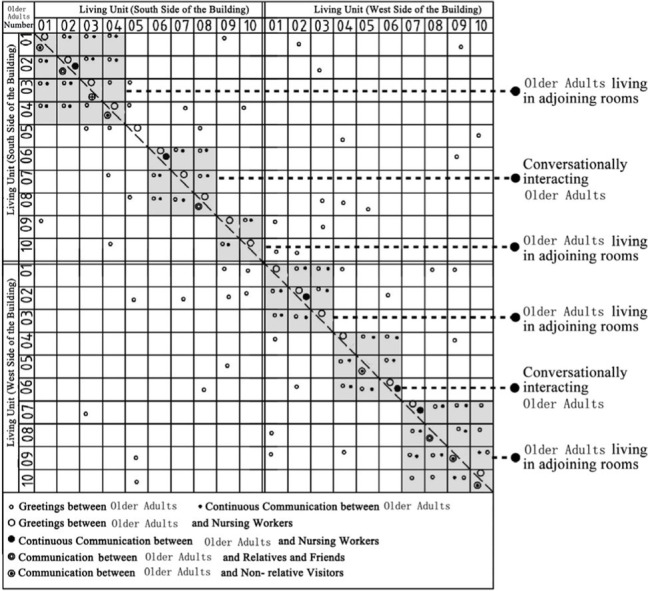	JP08 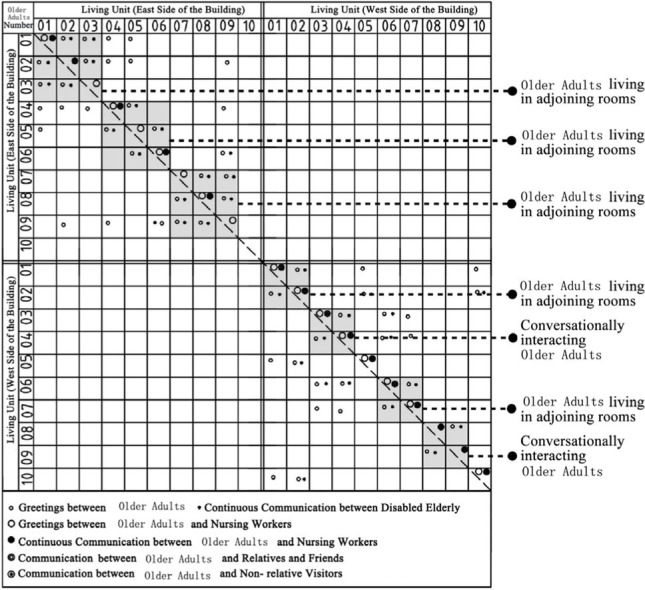

In DL01 and SY01, the point distributions of the communication behavior objects of the older adults individuals are relatively scattered. The objects of communication behavior are not concentrated in their respective living units. This shows that older adults in different living units exhibit transient communication behavior with each other. The reason is that the whole building of case DL01 does not adopt the design of a bedroom cluster. Through the cluster design, bedrooms of older adults can be centrally arranged in the southern part of the building, and the enclosed communication activity space can be centrally arranged in the northern part of the building. Although the whole building of case SY01 adopts the design of a bedroom cluster, there is no independent communication space for older adults in the living unit of the cluster, and only the public activity room is centrally designed at the corner of the building. The communication activity space in non clustered nursing units adopts a centralized design, resulting in relatively little continuous communication between older adults and their roommates, between older adults and nonrelative visitors, and between older adults and nursing workers in addition to the collective activities organized by the nursing home.

1. Scatter plot characteristics of the relationships between communication behavior objects on the same building floor based on behavior observations in group type nursing units.

In JP01 and JP08, the communication behaviors of older adults are concentrated in their own independent living units, and the frequency of continuous exchanges between various types of communication behaviors is relatively high. The reason for this phenomenon is that the space boundary of the living unit in the bedroom cluster of older adults is clear, and there is an independent and open common living space for older adults in the living unit. The daily activities of older adults, such as dining, games, entertainment, exercise and meeting with friends, are carried out in the living unit. Communication between older adults in different living units is rare.

#### 3.3.2 Investigation and characteristic analysis of the actual state in the field of communication behavior of the older adults

1. Scientific definition of the field of communicative behavior.

The definition of field in environmental behavior is a place where individuals or groups own or occupy an area and personalize it to meet certain needs. The field of communication behavior described in this paper refers to the field of behavior that guides the communication behavior object to form a behavior field that meets the communication psychological needs of older adults in the architectural physical space environment based on the communication psychological expectations of older adults. In the field of communication behavior, older adults make use of the architectural space or arrange the elements of the space environment to enhance their field to better meet the internal psychological needs of communication (Altaian and Rogoff, 1987; Takahashi Takashi and EBS Group, 2006; MASS Design Group, 2020; Jensen and Padilla, 2017; Mazuch, 2017).

2. Investigation and analysis on the field of communication behavior of the older adults in field investigation cases.

This paper uses the theory of interpenetration in environmental behavior to investigate and analyze the communication behavior of older adults in field investigation cases. Interpenetration theory emphasizes that behavior and the environment are dynamic systems in which they penetrate, influence and connect with each other (Altaian and Rogoff, 1987; Takahashi Takashi and EBS Group, 2006). The interpenetration process of behavior and the environment is manifested in the formation of a behavior field in an architectural space environment. In the design of communication spaces and environments for older adults in unit-type nursing home, attention should be given to the systematic integrity of multiple factors intertwined in architectural design and the subjectivity of users (Chaudhury et al., 2018). Therefore, by investigating the interpenetration between the communication behavior field and the communication space of older adults, the architect’s design expectation and the actual utilization of the architectural space can be connected, and the use of the communication space environment of older adults in unit-type nursing home can be further evaluated. If the overlap level of the communication behavior field and communication space is high, the architect’s design of the communication space meets the internal cluster communication needs of older adults. The communication behaviors of older adults interpenetrate with their communication space environment. In contrast, as the degree of overlap between the communication behavior field and the communication space of older adults decreases, the architect’s design of the communication space environment cannot meet the internal communication psychological needs of older adults. The results also reveal a deviation between the design of the original communication space environment and the actual use of older adults. (Figure 1).

This investigation of the communication behavior of older adults was carried out over 3 days. It was from 8:00 to 21:00. During this period, the communication behavior and place of the older adults individuals were observed at a fixed point at an interval of 10 min and recorded in the corresponding position of the floor plan. There was no specific size in the field of communication behavior, which reflects the spatial range required by older adults regarding communication psychology (Altaian and Rogoff, 1987; Takahashi Takashi and EBS Group, 2006; LaVela et al., 2016; Liddicoat et al., 2020). To embody the concept of this abstract field of behavior with psychological characteristics, this study uniformly used the gray circular logo to mark the field of communication behavior, and the circular size was determined according to the range of behavior activities of older adults in the field of communication behavior. (Table 3)

3. Investigation on the field of communication behavior of older adults in non clustered nursing units.

Case DL07: The functional training room, activity room and other communication spaces are located in the northern part of the building, and an enclosed and centralized design is adopted. Through behavior observation, it is found that the utilization rate of the above communication space is low. The overlap between the communication behavior field and communication space of older adults is low, and the design of the communication space in the northern part of the building fails to meet the psychological needs of older adults in terms of internal communication. The communication behavior field of older adults is mainly concentrated in the public space on the east and west sides of the patient. Older adults often stay in adjacent corridor spaces for communication. Sofa and plant bonsai are set in a small space at the end of the corridor on the east side of the building, which forms a small communication behavior field. Due to the lack of group division of bedrooms in architectural space design, there is a lack of order between the communication behavior activities of older adults, and the behavior activities will interfere with each other.

Design suggestion: A cluster design should be used for the bedrooms of older adults. At the same time, the communication space on the north side of the building is designed in an open way. In the public space on the east and west sides of the patient, where communication behavior frequently occurs, tables, chairs and other furniture are arranged to form a secondary communication space to effectively meet the internal communication needs of older adults and improve the utilization of communication space.

Case SY01: The activity room is centrally designed at the corner on the northeast side of the building as the communication space in the nursing home, which is also the main place for the formation of the communication behavior field of older adults. The communication behavior objects are mainly older adults living in the adjacent bedrooms around the activity room. Because the activity room at the turning point on the northeast side of the building is too far away from the bedroom at the end of the living unit, older adults living at the end of the living unit usually choose to engage in communication behavior in the small space at the end of the corridor on the west and south sides of the building. A common public bathroom is designed in the nursing home. When a large number of people are waiting for a bath, older adults choose to form a communication behavior field in the corridor space outside the public bathroom. Therefore, the overlap rate between the existing communication space in the nursing home and the communication behavior field of older adults is relatively low, and the design of the communication space environment cannot meet the psychological needs of older adults.

Design suggestion: an independent secondary communication space should be designed on the west and south sides of the building to meet the needs of older adults in the bedroom at the end of the corridor, and the communication crowd should be dispersed in the activity room at the turning point on the northeast side of the building. Meanwhile, secondary communication space around the public bathroom should be designed to meet the needs of waiting and resting before and after the bathing of older adults.

4. Investigation on the field of communication behavior of older adults in group type nursing units.

Case JP01: The communication behavior of older adults is relatively evenly distributed in each communication space. The overlap between the field of communication behavior and the communication space is relatively high. The existing communication space design meets the internal needs of older adults. The design of the common living space of older adults involves communication. At the same time, TV and seats are arranged in the small space at the end of the corridor on the north and west sides of the building, forming a secondary communication space carrying the field of communication behavior of the older adults. A rest space is designed in front of the public bathroom and laundry room to meet the communication needs of the older adults during the waiting period. Sofa is arranged in the rest and waiting space in front of the elevator in the northeast corner of the building, but due to the large flow of people, it interferes with the communication of the older adults, and there is no communication field in this space. The field of communication behavior of the older adults is mostly formed in the corridor space outside the functional training room.

Design suggestions: Seats should be flexibly arranged in the corridor space outside the functional training room to form a secondary communication space to meet the communication needs of older adults before and after physical exercise.

Case JP08: This case adopts a bedroom group design to form a living unit, and an independent living and communication space for older adults is designed in the living unit. Through behavioral observation, it is found that the overlap between the secondary communication space formed by the small space in the living unit on the west side of the building and the spontaneous and small-scale communication behavior field is greater than that on the east side, and the interpenetration relationship between behavior and environment is relatively better. The remaining space in front of the public bathroom and laundry room can better meet the occurrence of communication behavior and the formation of communication behavior field of the older adults. The design of the waiting space in front of the porch, the layout of furniture and the decoration of the wall also provide a small architectural space environment for small and scattered spontaneous fields of communication behavior. The adjacent small space around the common living space of older adults and the field of communication behavior also overlap greatly. The communication behavior field of older adults and the location of the common living space in the living unit on the east side of the investigated case building highly overlap. The design of a common living space for older adults better meets the functional, communication, psychological and practical needs of older adults. There is a good interpenetration relationship between the common living space and the communication behavior of older adults (Table 4).

## 4 Result

### 4.1 Comparative analysis on the characteristics of architectural space composition of field research cases

1. In non clustered nursing units (Building cases of nursing homes DL07 and SY01), usually focuses on designing communication spaces for older adults on the north side of buildings or at the turning point of building blocks, as shown in Table 1. This will lead to a long flow line between the bedrooms of older adults at the end of the corridor space and the communication activity space. The functions of architectural space are divided according to the content of communication activities. Communication activity spaces with different functions adopt an enclosed design, which leads to a lack of connection between spaces and a reduction in the utilization rate of communication activity space.
2. In group type nursing units (Building cases of nursing homes JP01 and JP08), usually adopts the spatial layout of bedroom clusters, as shown in Table 1. The living unit space formed by the cluster has clear boundaries, and the communication activity space is scattered in each living unit to meet the needs of older adults in the living unit. The communication activity space has spatial characteristics such as an open, composite function and decentralized layout, forming a common living space for older adults in daily life and creating a home living space atmosphere for older adults in the living unit (Pirinen, 2016).

### 4.2 Comparative analysis of communication characteristics of older adults in field research cases between group type and non clustered nursing units

1. There are relatively few objects of continuous communication behavior in non clustered nursing units (Building cases of nursing homes DL07 and SY01), the relevant empirical survey data for the study are shown in Table 7. The communication places are usually located in the corridor space and bedroom space, as shown in Table 3. There is a deviation between the environmental design of communication spaces and the actual use of older adults. In the field investigation of nursing home building cases DL01 and SY07, this study used the theory of mutual infiltration in environmental behavior to investigate and analyze the relationship between the social behavior of older adults and architectural space design (Altaian and Rogoff, 1987; Takahashi Takashi and EBS Group, 2006). Based on the theory of mutual infiltration between the environment and behavior (Figure 1), it is found through research that degree of overlap between the communication behavior field and the communication space of older adults is relatively low in nursing homes DL01 and SY07, the relevant empirical survey data for the study are shown in Tables 3, 4. So the field of communication behavior of older adults is not within the communication space designed by the architect (Table 3), who is based on his subjective experience, which leads to a reduction in the utilization rate of communication space, a lack of order between the communication behavior activities of older adults and interference between behavior activities.
2. There are relatively more objects of continuous communication behavior in group type nursing units (Building cases of nursing homes JP01 and JP08), the relevant empirical survey data for the study are shown in Table 7. Communication places are usually located in common living spaces in living units, as shown in Table 3. In the field investigation of nursing home building cases JP01 and JP08, this study used the theory of mutual infiltration in environmental behavior to investigate and analyze the relationship between the social behavior of older adults and architectural space design (Altaian and Rogoff, 1987; Takahashi Takashi and EBS Group, 2006). Based on the theory of mutual infiltration between the environment and behavior (Figure 1), it is found through research that degree of overlap between the communication behavior field and the communication space of older adults is relatively high in nursing homes JP01 and JP08. The behavior activities carried out in the communication behavior field of older adults involve interpenetration with the communication space environment. So the design of a communication space effectively meets the psychological needs of older adults, the relevant empirical survey data for the study are shown in Tables 3, 4. The actual utilization of the existing communication space by older adults fits well with the design expectations of architects.

### 4.3 Spatial environment design countermeasures based on the investigation of the real state of communication life of older adults

**Figure 2:**
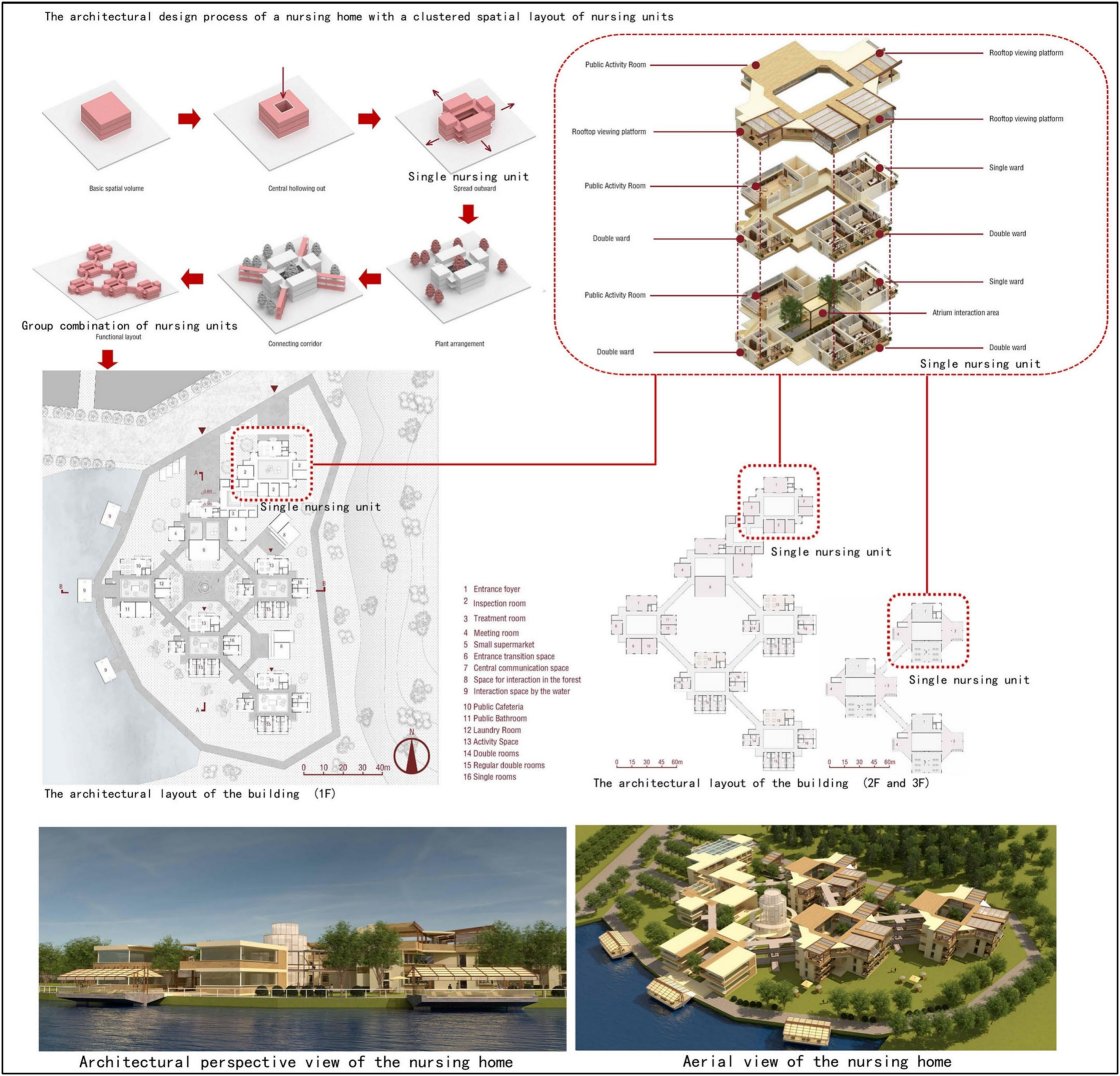
Trial design of a small scale cluster design adopted for the living units of unit-type nursing home.

1. A small-scale cluster design is adopted for the living units of the whole building (Figure 2): the bedroom cluster is used to make a living unit with clear spatial boundaries for older adults based on their physical conditions. In addition, an independent common living space should be designed as a carrier of communication behavior in the living unit to meet the daily communication needs of older adults. The design characterized by living units with clear spatial boundaries and independent and scattered common living space ensures order between the communication fields of older adults, which avoids interference between communication activities and facilitates the care, organization and management of older adults by nursing workers (Pirinen, 2016; Parke and Friesen, 2015).
2. An open communication space characterized by a family life atmosphere should be designed in living units: an open design is recommended for the communication space of older adults in a single living unit to ensure the accessibility of the space. Architectural elements such as walls, columns and doors, which hinder the use of communication space by older adults, should be weakened as much as possible to improve the utilization of common living space. Common living spaces should be equipped with the composite functions of cooking, dining, entertainment, games, exercise and so on to create a family life atmosphere, which can stimulate communication between older adults in many ways (Nakhodaeezadeh et al., 2017; Parke et al., 2017). Space environment elements such as tables and chairs, bookshelves, plants and light shelters that are easy to move should be used to flexibly divide common living spaces to form a rich and effective secondary communication space to meet the selectivity and control of older adults over communication space (Totaforti, 2018; Van Steenwinkel et al., 2017).
3. Space facilities equipped with urban life functions should be embedded between living units, connecting different living units with each other by using corridor spaces that have the street atmosphere of communication life. Small daily necessities such as supermarkets, cafes, teahouses, bookstores, flower shops, post offices and other public facilities with urban life functions are designed on both sides of the corridor space to promote the communication of older adults in different living units to avoid the separation of older adults from their original urban life (Carnemolla and Bridge, 2019; Fancourt and Finn, 2019). At the same time, small children’s day care space facilities can also be embedded between living units to organize interactive activities between older adults and children to effectively improve the communication life of older adults in nursing homes and promote the mental and physical health of older adults.

### 4.4 The theoretical and practical contributions of the study, and the value of this study for older adults’ care practice and policy implications

#### 4.4.1 Theoretical contribution

1. Enrich the theoretical system of older adults care.

This study delves deeply into the influence mechanism of the environment on the interaction behavior of the older adults, providing new empirical support for the theory of older adults care. It reveals how factors such as spatial layout, functional zoning, and communication space design specifically act on the social interaction, psychological state, and quality of life of the older adults, thereby enriching the research content on the relationship between architectural space design and the behavior of the older adults in the theory of older adults care.

2. Promoting interdisciplinary theoretical integration.

This research spans multiple disciplines including architecture, psychology, and behavioral science, achieving an organic integration of theories from various fields. By drawing on the spatial layout theory in architecture, the architectural psychology theory in psychology, and the theory of mutual infiltration of environmental behaviors, this study provides a new perspective and methodology for older adults care research.

3. Promoting innovation in research methodology.

Based on the theory of mutual infiltration of environmental behaviors, this research introduces new research methods. Through behavioral observation and quantitative analysis of the degree of mutual infiltration between space and behavior (Altaian and Rogoff, 1987; Takahashi Takashi and EBS Group, 2006), it aims to more accurately assess the impact of spatial layout on the interaction behaviors of the older adults. The innovations of these methodologies not only enhance the scientificity and accuracy of the research, but also provide new ideas for future geriatric care research.

#### 4.4.2 Practical contribution

1. Enhancing the social interaction behavior and quality of life of the older adults.

The spatial layout optimization design strategy of the nursing unit cluster proposed in this research result effectively promotes the social interaction behavior of the older adults by optimizing the spatial layout, enhancing the sense of belonging to the space, and providing diverse social venues. This not only helps alleviate the loneliness and depression of the older adults, but also enhances their life satisfaction and sense of happiness.

2. Optimizing the operational efficiency and service quality of nursing homes.

The optimization design strategy proposed in this research achievement effectively enhances the operational efficiency of nursing homes by rationally planning the scale of care units, optimizing care processes, and improving space utilization. Meanwhile, by providing a comfortable, safe and convenient living environment, this design has also improved the service quality of the nursing home and enhanced the sense of belonging and satisfaction of the older adults.

3. Promoting innovation in older adults care service models.

The research results on the optimized design of the spatial layout of nursing unit clusters have also promoted the innovation of older adults care service models. It encourages older adults care homes to pay more attention to the social and psychological needs of the older adults, and promotes the transformation of older adults care services from single physical care to comprehensive physical and mental care. The innovation of this service model not only helps improve the quality of life of the older adults, but also contributes to promoting the sustainable development of older adults care services.

4. Provide scientific basis for policy-making and industry standards.

The results of this study provide an important scientific basis for the government to formulate relevant older adults care policies, industry standards, as well as the construction and renovation of older adults care facilities. It helps promote the standardization, normalization and professionalization of older adults care services, and improve the service level and quality of the entire older adults care industry.

#### 4.4.3 The value of this study for older adults’ care practice

The spatial layout optimization design strategy of the nursing unit group proposed by this research result provides scientific support for older adults care practice by improving nursing efficiency, promoting the mental health of the older adults, ensuring safety and comfort, and demonstrating flexibility and sustainability. This model not only optimizes the care process, but also meets the emotional, social and self-actualization needs of the older adults through spatial design. It is an important development direction in the design of older adults care facilities.

1. Small-scale group-based spatial layout can enhance nursing efficiency and service quality.

Organizing living Spaces around nursing stations and public activity areas to form a nursing unit enables nursing staff to efficiently cover limited areas and reduce ineffective movement.

2. Clear functional zoning and optimized night duty.

Within the group, areas such as nursing, rehabilitation, and rest are divided to avoid overlapping flow lines. Nursing staff can quickly complete tasks such as daily care and health monitoring, reducing their work intensity. Adjacent groups can share nursing staff, saving labor costs while ensuring the quality of 24-h monitoring.

3. Promote the mental health and social participation of the older adults, and enhance their sense of belonging and social interaction.

Each group is equipped with a semi-private public living hall where the older adults frequently engage in activities, forming a stable social circle. Some group designs incorporate play areas or shared kitchens to facilitate communication among the older adults. The design of psychological healing Spaces, memory cafes, meditation corners, etc., combined with nostalgic decorations and natural sound effects, can soothe the emotions of the older adults.

#### 4.4.4 The research results have profound significance for policy-making and the optimization of the older adults care service system

1. Optimize the design standards of nursing homes to establish a foundation for formulating policies and architectural guidelines for elder care. This research aims to foster innovation in elder care service policies and models while enhancing the allocation of resources dedicated to older adults.

The spatial layout optimization strategy proposed by this research offers a quantifiable and replicable architectural design solution that enhances the older adult care service system. This model is aligned with policy demands aimed at proactively addressing population aging. By promoting standardization, supporting policy implementation, and optimizing resource distribution, it provides essential design standards, norms, and policies necessary for constructing a high-quality elder care service system.

2. Optimize the allocation of land and financial resources while promoting interdepartmental policy coordination.

The modular design approach for architectural spaces can significantly reduce renovation costs associated with existing nursing homes, allowing group-based spatial layouts to be extended to middle- and low-income elder care institutions. This facilitates broader coverage of elder care policies across diverse demographic groups. Furthermore, optimizing land use and financial resources through functional integration—such as combining nursing stations with public activity areas—can minimize transportation space requirements while improving land utilization rates. It is crucial to study the long-term operational costs associated with quantifiable spatial group designs in nursing homes—including labor savings and energy consumption reductions—to provide a solid basis for developing public subsidy policies for elder care as well as informed investment decisions.

### 4.5 The feasibility of the design strategy

#### 4.5.1 Design cost control strategy: modular design and resource integration of architectural space

1. Modular design reduces costs.

By adopting standardized group modules, the demand for customized construction can be reduced and the construction cost per bed can be lowered. For example, modular design can reduce the construction cost of a single bed and shorten the construction period at the same time.

2. Functional integration reduces space waste.

Integrating functions such as the nursing station, rehabilitation area, and public activity area into the core area of the group can reduce unnecessary Spaces such as corridors and traffic areas, lower the demand for building area, and thereby indirectly reduce land costs. After optimization, the space utilization rate can be improved and space waste can be reduced.

3. Flexibly adapt to low-budget scenarios.

The core care unit cluster space of the nursing home can be prioritized for construction. Subsequently, other functional areas can be gradually expanded based on the budget. Meanwhile, low-cost alternative solutions such as movable partitions and lightweight furniture are adopted to reduce the initial investment.

#### 4.5.2 Optimization of maintenance costs: the design of the nursing home adopts durable building materials and intelligent management

1. The design of the nursing home adopts a group-based spatial layout of care units, which can standardize the maintenance process.

Formulating regional maintenance manuals, clearly defining the cleaning and maintenance standards for public areas, care areas and residential areas, can reduce repetitive work and improve maintenance efficiency.

2. The nursing unit adopts intelligent management to reduce labor costs.

By installing temperature and humidity sensors, intelligent lighting systems and other automated environmental control systems, as well as Internet of Things sensors for key equipment such as elevators and air conditioners, fault early warning and remote monitoring can be achieved, reducing the frequency of manual inspection and maintenance, and lowering the cost of human maintenance.

3. The basic care units in nursing homes are designed with durable building materials.

Choosing materials such as wear-resistant and easy-to-clean PVC floor tiles and antibacterial wall coatings can reduce the frequency of long-term maintenance and lower maintenance costs. For instance, the unit price of PVC floor tiles is 40% lower than that of stone tiles, and their durability is comparable.

#### 4.5.3 Low budget adaptability: policy support and community collaboration

1. Integration and sharing of community resources.

Cooperate with the surrounding communities to open the rehabilitation areas, activity rooms and other facilities within the nursing home cluster to the communities during off-peak hours, and share the facility costs. In addition, college student volunteers and mutual aid groups for young older adults people can also be introduced to participate in the organization of social activities to reduce the labor cost of full-time social workers.

2. Innovation in low-cost operation models.

Intergenerational integration service models can be explored, such as introducing college student volunteers and mutual aid groups for young older adults people to participate in the organization of social activities, and replacing part of the bed fees with services. At the same time, cooperate with surrounding manufacturers to purchase older adults-friendly furniture and consumables to reduce logistics and procurement costs.

3. Balance short-term investment with long-term benefits.

Through modular design, functional integration and low-cost materials, the construction cost of a single bed in a group layout can be controlled within 1.1 times that of the traditional mode, and the initial investment can be further reduced through policy subsidies. Improving the interaction behavior of the older adults can reduce the depression rate, enhance the efficiency of care, and indirectly lower the long-term operating costs.

## 5 Conclusion

In non clustered nursing units research, centralized and enclosed designs are adopted because the space functions are limited while the communication space of older adults is designed, which leads to a mismatch between the communication behavior field of older adults and the communication space designed based on the architect’s subjective experience. Therefore, the utilization rate of the communication space is low, and the communication behavior activities of older adults interfere with each other. In group type nursing units research, the communication space of older adults is scattered in the living unit formed by the bedroom cluster. The composite functional, open and family-style design of the communication activity space meets the psychological needs of older adults. There is a good interpenetration relationship between the communication behavior field and the communication space of older adults. There are several spatial environment design methods based on the investigation of the real state of the communication life of older adults:

First, a small-scale cluster design should be adopted for the living units of the whole building. The older adults individuals were classified according to their physical health to make a living unit with clear spatial boundaries for the older adults individuals based on their physical conditions;

Second, a common communication space with an open and family atmosphere style in the living unit can function as the main place for the living and communication of older adults. Environmental elements such as furniture, potted plants and light partitions can be used to flexibly divide the common communication space to form a secondary communication space connected with each other;

Finally, space facilities equipped with urban life functions should be embedded between living units to promote the socialized communication life of older adults.

### 5.1 The limitations of the study and future research prospects

Research on the architectural space layout and interaction space design of future nursing homes must comprehensively consider the diverse factors that influence the potential interaction preferences of older adults individuals. These factors include regional cultural differences, socioeconomic status, educational levels, and cognitive variations. For instance, there are significant disparities in older adults care concepts and facility foundations between urban and rural areas as well as across different regions. Additionally, regional cultural differences can significantly impact the interaction space preferences of older adults. The findings from this research will facilitate a more accurate response to the varied needs of the older adults population through dynamic adaptive adjustments and multivariate collaborative analyses in designing communication spaces within nursing homes.

1. The design of communication spaces in nursing homes should establish guidelines that incorporate elements such as regional cultural identification systems and designated areas for traditional regional cultural festival activities. This approach aims to enhance the adaptability of communication spaces to local cultures.
2. Considering the diversity among older adults populations regarding socioeconomic status, educational attainment, and cognitive abilities, it is essential to develop a hierarchical and classified framework for interaction spaces within nursing homes. Such designs should align with the dynamic functions required by architectural layouts. For example, furniture and partitions within these interaction spaces ought to feature movable and foldable designs that allow for rapid reorganization according to varying needs.
3. The nursing home adopts a “modularization + variability” design strategy. While using standardized care unit group modules, it introduces dynamic adaptive interaction space design to build Spaces that are adaptable to the life cycle of the older adults. For instance, it uses modular and expandable architectural space design and reserves interfaces for functional space expansion. In the future, appropriate architectural Spaces can be added according to the physical health differences and potential communication preferences of the older adults population.

## Data Availability

The original contributions presented in the study are included in the article/supplementary material, further inquiries can be directed to the corresponding author/s.

## References

[ref1] Altaian I. Rogoff B. (1987). “World views in psychology: trait, interactional, organismic, and transactional perspectives” in Handbook of environmental psychology. eds. Stokols D. Altman I., vol. 1 (New York: Wiley), 1–40.

[ref2] Bravo-Farré L. Tamashiro-BEPPU M. Li G. L. (2012). A study of two elderly housing and their design strategies in Catalonia, Spain. Time Architec. 6, 48–53. doi: 10.13717/j.cnki.ta.2012.06.013

[ref3] Carnemolla P. Bridge C. (2019). Housing design and community care: how home modifications reduce care needs of older people and people with disability. Int. J. Environ. Res. Public Health 16:1951. doi: 10.3390/ijerph16111951, PMID: 31159396 PMC6604004

[ref4] Chaudhury H. Cooke H. A. Cowie H. Razaghi L. (2018). The influence of the physical environment on residents with dementia in long-term care settings: a review of the empirical literature. Gerontologist 58, e325–e337. doi: 10.1093/geront/gnw259, PMID: 28329827

[ref5] Fancourt D. Finn S. (2019). What is the evidence on the role of the arts in improving health and well-being? A scoping review. Copenhagen: WHO Regional Office for Europe. 32091683

[ref6] Feddersen E. Ludtke I. Dwight M. B. Dtke I. L. (2009). Living for the elderly: A design manual (design manuals). Switzerland: Birkhauser Architecture.

[ref7] Gobbens R. van Assen M. (2018). Associations of environmental factors with quality of life in older adults. Gerontologist 58, 101–110. doi: 10.1093/geront/gnx051 28510656

[ref8] Hu M. (2004). Creating safe and comfortable bathroom spaces - consideration for the elderly and mobility impaired. Hua zhong Architec. 4, 70–71. doi: 10.13942/j.cnki.hzjz.2004.04.026

[ref9] Huang H. Zhen D. J. Zhang F. (2001). Centerline international senior village design experience. Archit. J. 11, 44–46. Available at: https://kns.cnki.net/kcms2/article/abstract?v=db-EbSzo3oj4BTx-FCu1KVNazjnyvCN8OdHk046QGxnnu_88si_pwyp_YYYVSKN3MiVlKrr7lZFxyXKqk-5A2IOUNe2FUOvMIENAZmy7LKKFqejq13lYyRsEMkGZgTQmOj2K8VWRK0Z32PHmse6qF38ElK6aZCKw2ozM1HUcmzL8dWGdc6cA6A==&uniplatform=NZKPT&language=CHS

[ref10] Jensen L. Padilla R. (2017). Effectiveness of environment-based interventions that address behavior, perception, and falls in people with Alzheimer’s disease and related major neurocognitive disorders: a systematic review. Am. J. Occup. Ther. 71:7105180030p1-7105180030p10. doi: 10.5014/ajot.2017.027409, PMID: 28809653

[ref12] LaVela S. L. Etingen B. Hill J. N. Miskevics S. (2016). Patient perceptions of the environment of care in which their healthcare is delivered. Health Environ. Res. Design J. 9, 31–46. doi: 10.1177/1937586715610577, PMID: 26597101

[ref13] Li Q. L. Li B. (2012). The living behavior mode in elderly facilities. Time Architect. 6, 30–36. doi: 10.13717/j.cnki.ta.2012.06.010

[ref14] Liddicoat S. Badcock P. Killackey E. (2020). Principles for designing the built environment of mental health services. Lancet Psychiatry 7, 915–920. doi: 10.1016/s2215-0366(20)30038-9, PMID: 32171432

[ref15] Liu Y. J. (2000). Aging society and senior housing. Archit. J. 8, 24–26. Available at: https://kns.cnki.net/kcms2/article/abstract?v=db-EbSzo3ohEXtY7fn1oPvbPgYTBDqxkBfR1f3gFHXxkr-S54uv462z_cdCuWPzVRuSgZu-hA3iG82pUvpla0GTdV7KhwEiymdjQn_4FCjp0ZPt4XDg9ujnfN-YNSJx-G1PBgl39-Y8IMiAnycM87xAYBmXbb87KQ7aBBfaGr6kGL2kF2kE2rA==&uniplatform=NZKPT&language=CHS

[ref16] Lu W. (2005). Building compound community public space for the aged. Huazhong Architect. 5, 20–23. doi: 10.13942/j.cnki.hzjz.2005.05.037

[ref17] Lu W. Zhou B. Wang S. Y. Wang H. Y. Zhou Q. Li T. L. (2011). Study on public space of senior houses: taking institutional senior houses in Dalian and Shenyang as examples (2). Archit. J. S1, 160–164. Available at: https://kns.cnki.net/kcms2/article/abstract?v=db-EbSzo3ohpbe-cUjFksI_73aMIzlQ2gNLWwXSIBJgkbKKDfYP3b_Y_RSAirrSdTZeh9vjha-TGBSmHuockmw2zsMW1X4YTZsHpsIb9gdVnQNCVDUmhdU2JofMbjBjIhxoJFLLB9JzfR0b5ZdSGxkSqyz82RfhddzZin8tipDD2Gz9PfRXeOw==&uniplatform=NZKPT&language=CHS

[ref18] Ma H. Zhao G. Y. (2004). The design research on the apartments for the elderly. Huazhong Architect. 22, 52–5458. doi: 10.13942/j.cnki.hzjz.2004.01.019

[ref19] MASS Design Group. (2020). Our mission is to research, build, and advocate for architecture that promotes justice and human dignity. Available online at: https://massdesigngroup.org/about Retrieved (Accessed December 13, 2021)

[ref20] Mazuch R. (2017). Salutogenic and biophilic design as therapeutic approaches to sustainable architecture. Archit. Des. 87, 42–47. doi: 10.1002/ad.2151

[ref21] Nakhodaeezadeh M. Jafarabadi M. A. Allahverdipour H. Matlabi H. Dehkordi F. R. (2017). Home environment and its relation with quality of life of older people. J. Hous. Elderly 31, 272–285. doi: 10.1080/02763893.2017.1280583

[ref22] Parke B. Boltz M. Hunter K. F. Chambers T. Wolf-Ostermann K. Adi M. N.. (2017). A scoping literature review of dementia-friendly hospital design. Gerontologist 57, e62–e74. doi: 10.1093/geront/gnw128, PMID: 27831481

[ref23] Parke B. Friesen K. (2015). Code plus: Physical design components for an elder friendly hospital. 2nd Edn. Canada: Fraser Health.

[ref24] Peavey E. Vander Wyst K. B. (2017). Evidence-based design and research-informed design: what’s the difference? Conceptual definitions and comparative analysis. HERD 10, 143–156. doi: 10.1177/1937586717697683, PMID: 28349729

[ref25] Pirinen A. (2016). Housing concepts for and by the elderly: from subjects of design to a design resource. J. Hous. Elderly 30, 412–429. doi: 10.1080/02763893.2016.1224792

[ref26] Takashi T. EBS Group (2006). Environment-behavior studies’ data tools for spatial design. Beijing, China: China Architecture & Building Press.

[ref27] Totaforti S. (2018). Applying the benefits of biophilic theory to hospital design. City Territ. Archit. 5, 2–9. doi: 10.1186/s40410-018-0077-5

[ref28] Van Steenwinkel I. Dierckx de Casterlé B. Heylighen A. (2017). How architectural design affords experiences of freedom in residential care for older people. J. Aging Stud. 41, 84–92. doi: 10.1016/j.jaging.2017.05.001, PMID: 28610759

[ref29] Wang X. M. Yin H. L. Ma T. (2013). Analysis on the design theory and cases of welfare facilities for the elderly in Japan. Beijing, China: China Architecture & Building Press.

[ref30] Xia Y. T. (2013). Study on influence factors of city endowment facilities layout: taking endowment facilities planning of central district in Kunming as an example. Huazhong Architecture 6, 148–152. doi: 10.13942/j.cnki.hzjz.2013.06.035

[ref31] Xiao Y. (2017). The study of problems in long-term care services for the disabled elderly in China. Beijing, China: China Social Sciences Press.

[ref32] Zhong L. Zhang Y. L. Zhou Y. M. (2017). Design and type research on bathroom in elderly facilities. Architect. J. 4, 100–104. Available at: https://kns.cnki.net/kcms2/article/abstract?v=db-EbSzo3ohXczYGE4vgAP9EJ0VWZFoacpSBiAUd2BhulCv6P7CHJOukqx2Dp8RrKA_2VP9AkUSJsghY7rl2sznQejulguIeBLu8TqlwIdqJTK16Y7ThLCxF6IjXzeGIpWulwpHjPJ-aC079QAE-k6nCKGPp7vpe65sSG5GV560t3ZqfLtUffQ==&uniplatform=NZKPT&language=CHS

[ref33] Zhou Y. M. Chen X. Q. Lin J. Y. Lin J. Y. (2011). Living for the elderly. Beijing, China: China Architecture & Building Press.

[ref34] Zhou B. Wang H. Y. Lu W. Li T. L. (2013). Comparative study on the characteristics of spatial perception experience of eldercare building in China and Japan. Archit. J. S2, 66–71. Available at: https://kns.cnki.net/kcms2/article/abstract?v=db-EbSzo3oh01lM7dTDBJFvkNHvJKDtiBN5NWchVqUdc1qHd6kq-LvKDZuY-ZAwsRQlH1P73xaAKAxmjVpg9RUco_v2Op2ruARe4h1_Rli7h8eFeZ0QUxuQH5NQgyUC76hhUJOAlM7LAc5-Dxw_ULFFLnNQiWPt14JiyqqTPaAHvAeIhEhZ-qg==&uniplatform=NZKPT&language=CHS

